# OtoVCE: a mechanism-aware language-model evidence layer for hereditary hearing-loss variant interpretation

**DOI:** 10.3389/fgene.2026.1880490

**Published:** 2026-08-12

**Authors:** Shaopei Ye, Lan Wang, Peng Chen

**Affiliations:** 1 Faculty of Biology, University of Barcelona, Barcelona, Spain; 2 Department of Otorhinolaryngology Head and Neck Surgery, Chengdu Fifth People’s Hospital, Chengdu, Sichuan, China; 3 Department of Otorhinolaryngology Head and Neck Surgery, The Second People’s Hospital of Meishan City, Meishan, Sichuan, China

**Keywords:** ACMG/AMP variant interpretation, clinical decision support, hereditary hearing loss, large language model, mechanism-aware prompting

## Abstract

**Background:**

Missense variants in genes implicated in hereditary hearing loss are frequently returned to clinicians as variants of uncertain significance. *In silico* predictors and rule-based ACMG/AMP frameworks each capture only a fraction of the required evidence, and neither directly accesses case-level and functional evidence reported in the published literature.

**Methods:**

We developed OtoVCE, a four-stage framework that places a large language model inside a calibrated ACMG/AMP rule engine as a structured evidence extractor rather than an end-to-end classifier. Rule-encodable evidence—six *in silico* missense predictors, gnomAD allele frequencies, UniProt domain annotations, and a hearing-loss-specific protein language model with a pathogenicity head and a mechanism head—is aggregated by the ClinGen Hearing Loss VCEP rule set. For variants that remain uncertain, OtoVCE retrieves the literature from five sources and prompts the language model using the Brnich functional evidence rubric, with the predicted disease mechanism as context, returning PS3, BS3, and PS4 strength assignments with PubMed-verified citations. Rule-based and language-model-derived strengths are then combined into a posterior probability mapped to the five ACMG/AMP classes.

**Results:**

On a held-out post-2024 ClinVar cohort (N = 1,885), OtoVCE achieved an area under the receiver-operating curve (AUC) of 0.997 and a sensitivity of 93.6% at a 1% false-positive rate. Performance was preserved across an *OTOF* gene-leave-out cohort (AUC = 0.993), the external Deafness Variation Database (AUC = 0.917), and the protein-language-model training cohort itself, with model-derived rules disabled (AUC = 0.976), excluding training-set memorization. A paired A/B comparison (N = 1,930) attributed the literature evidence contribution to the mechanism cue: 7.1-fold more cited identifiers, 11.6-fold more quantitative evidence, and diagnostic strength scores (paired-bootstrap ΔAUC +0.18). OtoVCE identified 211 of 2,389 uncertain variants as one piece of supporting evidence short of likely pathogenic at 98.1% precision, agreed with ClinGen-VCEP curation at Cohen’s κ = 0.57, and verified 100% of 3,185 cited PubMed identifiers.

**Conclusion:**

A literature-grounded, mechanism-aware language-model evidence layer can serve as a complementary component within a calibrated ACMG/AMP workflow for clinical variant interpretation in hereditary hearing loss.

## Introduction

Sensorineural hearing loss is among the most genetically heterogeneous common disorders. More than 150 genes have been implicated in its non-syndromic and syndromic forms, and tens of thousands of hearing-loss-associated missense variants are cataloged in the Deafness Variation Database (Azaiez et al., 2018) and ClinVar (Landrum et al., 2018). In response to this interpretive burden, the ClinGen Hearing Loss Variant Curation Expert Panel has published a disease-specific elaboration of the ACMG/AMP framework (Richards et al., 2015; Strande et al., 2017; Oza et al., 2018), codifying the evidence required for a confident clinical classification. Despite this, a substantial fraction of newly observed missense variants in clinical sequencing remains classified as variants of uncertain significance (Shearer et al., 2017; Sloan-Heggen et al., 2016). The clinical consequences are direct: families complete exome sequencing without a definitive diagnosis; eligibility for emerging gene therapies, such as the AAV1-hOTOF program for autosomal–recessive deafness type 9 (Lv et al., 2024), cannot be confirmed at the laboratory bench, and per-variant curation to a five-class call scales poorly with the throughput of routine sequencing.

Three families of tools currently address components of this bottleneck, each with distinct strengths and limitations. Computational missense predictors—including REVEL (Ioannidis et al., 2016), AlphaMissense (Cheng et al., 2023), EVE (Frazer et al., 2021), ESM1b (Brandes et al., 2023), CADD (Rentzsch et al., 2019), and PrimateAI (Sundaram et al., 2018)—rank variants on a continuous pathogenicity scale. A recent ClinGen calibration study placed these scores on a likelihood-ratio scale compatible with the PP3 and BP4 evidence rules (Pejaver et al., 2022), but each predictor returns a single numerical value without the literature context or an explicit categorical recommendation. Rule-based ACMG/AMP frameworks such as InterVar (Li and Wang, 2017) and VarSome (Kopanos et al., 2019) implement the published specifications, return a five-class call, and document supporting evidence. However, they are restricted to evidence that can be encoded as a programmatic rule and therefore exclude most of the case-prevalence and functional evidence reported in the published literature. Single-shot classifications by foundation language models, which prompt the model to label a variant directly, have, in principle, accessed that literature (Brown et al., 2020; Singhal et al., 2023; Thirunavukarasu et al., 2023) but produce unstructured output with citations that are difficult to verify. In head-to-head benchmarking on hearing-loss variants, such single-shot classifications consistently underperform calibrated predictors and rule engines (Supplementary Figure S4A). No single tool family currently provides a complete clinical-grade pipeline.

The unmet need is therefore not predictive accuracy but the integration of predictor signal with the case-level and functional evidence required by the ACMG/AMP framework. We hypothesized that this integration could be achieved by repurposing a large language model as a structured evidence extractor rather than as a classifier (Lala et al., 2023; Ouyang et al., 2022). In this role, the model evaluates the literature retrieved from explicit sources and returns ACMG/AMP-graded evidence under a published functional-evidence rubric (Brnich et al., 2020). Its output is then incorporated into a Bayesian rule aggregator (Tavtigian et al., 2018) as Brnich-graded PS3, BS3, and PS4 strengths with verifiable PubMed citations. We further reasoned that extraction would be improved by providing disease-specific context to the model. In particular, supplying the predicted disease mechanism—derived upstream from a hearing-loss-specific protein language model with a mechanism head—should direct the model to the relevant passages in the retrieved literature and yield strength assignments calibrated to the rubric rather than to model confidence.

In this study, we describe OtoVCE, a four-stage framework that combines the ClinGen Hearing Loss VCEP rule engine with a mechanism-aware language-model evidence layer that operates over five literature sources under a PubMed-verified citation audit. We evaluate the framework on four cohorts of pre-classified hearing-loss missense variants, test whether the mechanism cue is causally responsible for the additional evidence the model extracts, and characterize both the framework’s clinical actionability and its remaining failure modes. Our findings do not displace existing *in silico* predictors or rule engines; rather, they indicate that a language-model evidence layer can serve as a complementary component within a calibrated ACMG/AMP workflow.

## Materials and methods

### Cohort construction

We assembled four cohorts of pre-classified hearing-loss missense variants, each designed to control a specific source of training–test contamination.

The post-2024 ClinVar cohort (N = 1,885) comprised ClinVar-curated variants (Landrum et al., 2018) with a *LastEvaluated* date strictly later than 1 January 2024. Any earlier ClinVar entries for the same variant were excluded so that no cohort variant was observed during framework development. The protein language model was trained only on ClinVar variants curated before this cutoff. The cohort was held out for the entire development process and serves as the primary clinical benchmark.

The *OTOF* cohort (N = 205) comprised every ClinVar missense variant in *OTOF*. The entire gene was withheld from protein-language-model training, isolating gene-level generalization from the temporal cohort separation (Lv et al., 2024).

The DVD cohort (N = 2,835) is the strong-evidence subset of the Deafness Variation Database (Azaiez et al., 2018), comprising variants with at least one curated citation and consistent classification across sources. It serves as an external benchmark of curatorial provenance distinct from the ClinVar-derived cohorts.

The protein-language-model training cohort (N = 8,096) consists of the variants used to train the protein language model. We evaluated the framework on this cohort with the two ACMG rules sourcing signal from that model disabled. Any residual advantage in this cohort must, therefore, originate from the rule engine or the literature-evidence layer rather than from memorized training labels.

Variants appearing in more than one cohort were tracked using a (gene, protein-change) tuple, with gene symbols following the HGNC nomenclature (Tweedie et al., 2021); cohort-specific metrics were computed on the corresponding variant set. Per-tool sample sizes in Table 1 reflect each predictor’s coverage of the relevant cohort. AlphaMissense (Cheng et al., 2023) scores were unavailable for the DVD cohort, and that predictor was therefore excluded from common-cohort analyses involving that cohort.

**TABLE 1:** Tool comparison across the four validation cohorts. Per-tool sample size, area under the receiver-operating curve (AUC) with bootstrap 95% confidence interval, and sensitivity at false-positive rates of 1% and 5%.

Cohort	Tool	N	AUC [95% CI]	Sens @ FPR = 1%	Sens @ FPR = 5%
post-2024 ClinVar	OtoVCE	1,885	0.997 [0.996–0.998]	93.6%	97.0%
post-2024 ClinVar	HL-PLM	1,869	0.993 [0.990–0.995]	85.1%	97.2%
post-2024 ClinVar	REVEL	1,503	0.965 [0.957–0.974]	66.5%	84.8%
post-2024 ClinVar	AlphaMissense	1,530	0.952 [0.940–0.962]	45.4%	78.5%
post-2024 ClinVar	CADD	1,874	0.941 [0.932–0.950]	47.3%	69.4%
post-2024 ClinVar	EVE	865	0.932 [0.916–0.947]	50.3%	67.2%
post-2024 ClinVar	ESM1b	1,507	0.919 [0.903–0.933]	35.4%	67.8%
post-2024 ClinVar	PrimateAI	1,521	0.859 [0.841–0.879]	14.7%	41.2%
*OTOF*	OtoVCE	205	0.993 [0.983–0.999]	66.4%	97.3%
*OTOF*	HL-PLM	205	0.977 [0.954–0.994]	29.2%	82.3%
DVD strong	OtoVCE	2,835	0.917 [0.906–0.929]	34.3%	66.1%
DVD strong	HL-PLM	2,815	0.899 [0.886–0.911]	18.4%	58.5%
PLM-train (rules off)	OtoVCE	8,096	0.976	72.5%	—
PLM-train (rules off)	REVEL	8,096	0.942	55.8%	—
PLM-train (rules off)	AlphaMissense	8,096	0.941	41.6%	—

Full per-tool numbers, including bootstrap 95% confidence intervals for every cohort, are provided in the Zenodo deposit accompanying this manuscript DOI: 10.5281/zenodo.21245197.

### Multi-source enrichment

For each variant, we integrated six independent sources of evidence: Deafness Variation Database curation (Azaiez et al., 2018); myvariant.info cached scores from AlphaMissense (Cheng et al., 2023) and REVEL (Ioannidis et al., 2016)/CADD (Rentzsch et al., 2019) as a redundancy layer; gnomAD v4 allele frequencies and gene-level constraint statistics (Chen et al., 2024); UniProt functional-domain annotations (UniProt Consortium, 2023); the ClinVar hereditary hearing-loss subset as a reference table for PS1 and PM5 (Landrum et al., 2018); and a hearing-loss-specific multi-task protein language model trained on (gene, protein-change, label) triplets drawn from the ClinVar hearing-loss subset before the 1 January 2024 cutoff. The protein language model returns a pathogenicity probability and a mechanism-head output distinguishing loss-of-function, dominant-negative, trafficking-defect, and transport-loss-of-function modes, together with a continuous estimate of the hearing-threshold shift (in decibels) associated with the variant. A “clean-predictors” pass refetched REVEL, AlphaMissense, EVE (Frazer et al., 2021), ESM1b (Brandes et al., 2023), PrimateAI (Sundaram et al., 2018), CADD, phyloP, and GERP scores when cached values were stale or missing.

### ACMG/AMP rule engine

The rule engine implements the ClinGen Hearing Loss Variant Curation Expert Panel specifications (Oza et al., 2018) across 17 rules. The pathogenic-direction rules comprise the ACMG/AMP very-strong (PVS1), strong (PS1), moderate (PM1, PM4, and PM5), and supporting (PM2 at supporting strength and PP3) criteria. Two hearing-loss-specific extensions of the framework supplement these. A phenotype-mechanism rule (derived from PP4) fires when the protein-language-model mechanism head matches the inheritance pattern. A supporting-evidence rule fires when the protein-language-model pathogenicity head exceeds a calibrated threshold. The benign-direction rules comprise BA1, BS1, BS2, BP4, BP7, and a benign phenotype-mechanism rule symmetric to the pathogenic extension. Each fired rule returns a strength on a four-level scale (*supporting*, *moderate*, *strong*, and *very strong*), which is mapped to a likelihood ratio in the Bayesian aggregation step. No language-model call is made at this stage; the rule output is deterministic.

### Mechanism-aware language-model evidence layer

For variants whose preliminary classification was uncertain, the evidence layer was run in two steps. In the first step, each variant was matched against a manually curated whitelist of hearing-loss functional assays. If an exact variant-level match was identified, PS3 and BS3 were assigned deterministically from the whitelist entry without invoking the language model.

**FIGURE 1:**
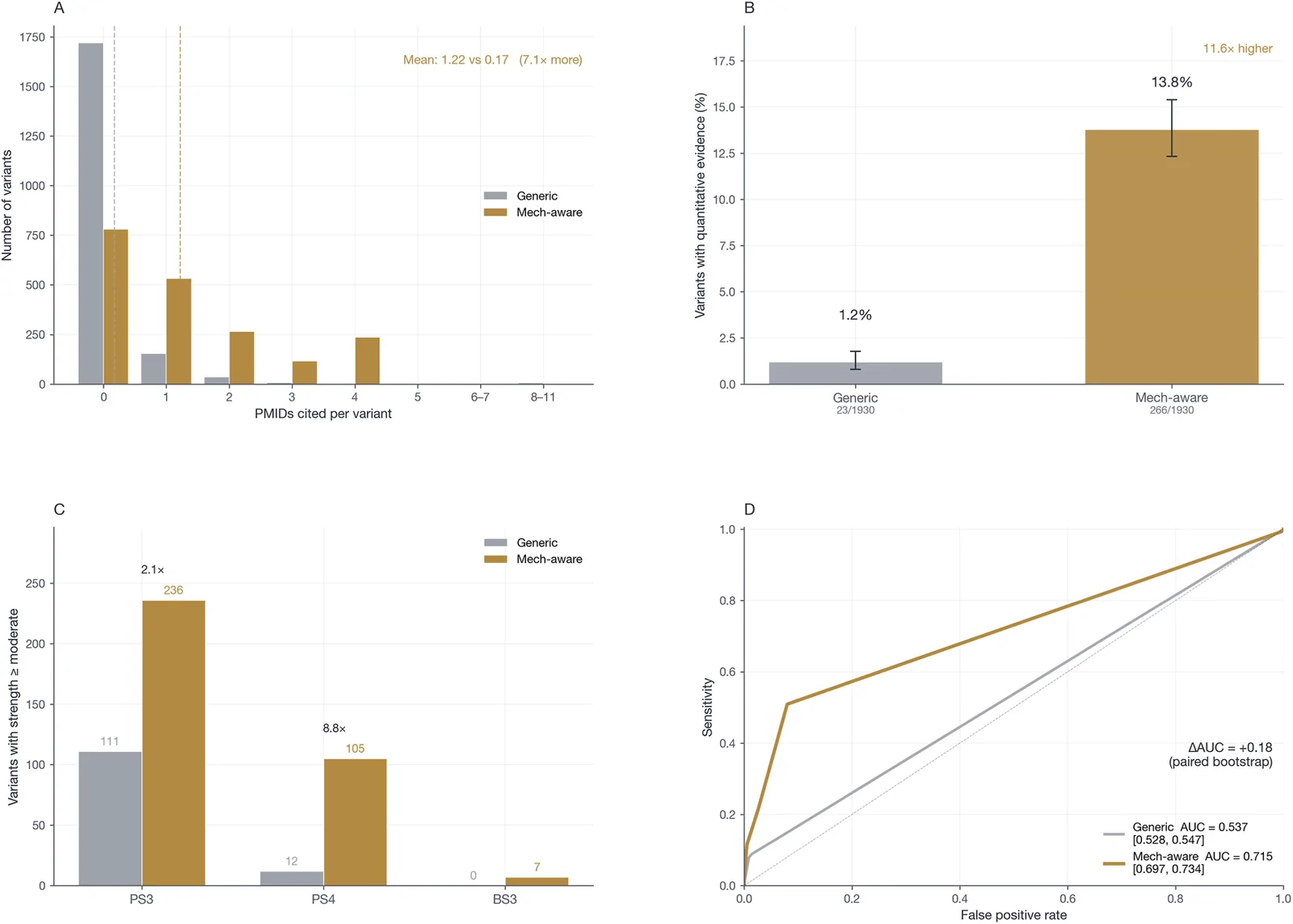
Mechanism-aware language-model evidence extraction (paired N = 1,930). Each variant was evaluated using the language model twice with the identical retrieved literature, once under the *mechanism-aware* prompt (protein-language-model mechanism prediction supplied as context) and once under the *generic* prompt (mechanism context removed; Brnich rubric otherwise identical). **(A)** Distribution of PubMed identifiers cited per variant. **(B)** Proportion of variants for which the model extracted quantitative evidence, with Wilson 95% confidence intervals. **(C)** Counts of variants assigned a strength of moderate or stronger for the PS3, BS3, and PS4 evidence types. **(D)** A composite per-variant strength score (PS3 + PS4 numeric strength minus BS3 numeric strength) as a stand-alone classifier of pathogenicity; areas under the curve compared by 1,000-resample paired bootstrap.

In the second step, the literature was retrieved from five complementary sources: a manually curated hearing-loss-gene whitelist, the variant’s Deafness Variation Database citations, LitVar 2 keyed on the variant rsID (Allot et al., 2023), PubMed abstracts queried with the gene symbol and HGVS-protein string (den Dunnen et al., 2016), and PubMed Central full-text passages. Retrieved articles were deduplicated using the PubMed identifier and passed to a large language model (DeepSeek-V3, model identifier deepseek-chat, accessed via the DeepSeek public API; DeepSeek-AI, 2024) under the Brnich functional-evidence rubric (Brnich et al., 2020). The model prompt included the predicted mechanism as context (the top-scoring mechanism among loss-of-function, dominant-negative, and trafficking-defect); the generic-prompt control used in Figure 1 omitted this context but was otherwise identical in retrieval, model, and rubric. The model returned a structured record containing per-evidence strengths (PS3, BS3, and PS4) on the same five-level scale used by the rule engine, along with the list of PubMed identifiers cited as supporting evidence. Because pathogenic and benign functional findings map to the opposing PS3 and BS3 strengths, conflicting reports for the same variant are represented as graded strengths in opposite directions rather than reconciled prior to aggregation. Under the Brnich rubric, each strength is conditioned on assay validity, controls, and replication, so a methodologically weaker report receives a lower strength and contributes correspondingly less weight. These opposing strengths are subsequently combined in the Tavtigian Bayesian framework (see *Bayesian aggregation and final classification*), where PS3 and BS3 enter as reciprocal likelihood ratios whose logarithms are summed; opposing evidence therefore partially cancels, while the net direction and magnitude are preserved rather than being averaged away. The PubMed identifiers cited as supporting evidence were retained in the evidence record, ensuring that every contributing report remained auditable by a human curator. Each cited identifier was verified against NCBI E-utilities, and its PubMed publication date was checked. Identifiers that did not resolve were flagged as unverified and removed from the evidence record. Identifiers with publication dates later than the temporal cutoff were retained but flagged for downstream audit so that any post-cutoff leakage into the rule engine output could be tracked (Supplementary Figure S1B). Because the model contributes only rubric-graded strengths anchored to E-utilities-verified citations rather than a class label, the literature encountered during the base model’s pre-training cannot enter the rule-engine output as a classification: only a resolvable citation supporting an extractable functional result propagates.

### Mechanism-head validation and sensitivity to mechanism error

To validate the protein-language-model mechanism head independently of the classification task, we assembled a gold-standard set of 137 hearing-loss missense variants with mechanism assignments curated from wet-laboratory functional studies (Wasano et al., 2020 for *SLC26A4*, published *GJB2* functional literature, and deep mutational scans of *KCNQ4* and *MYO7A*). Each variant’s top-scoring mechanism-head class was compared with its curated mechanism; we report overall top-1 accuracy with a bootstrap 95% confidence interval, the four-class confusion matrix (Supplementary Figure S7A), and accuracy at the loss-of-function-type-versus-dominant-negative granularity consumed by the phenotype-mechanism (PP4-derived) rule. To quantify the sensitivity of the final classification to mechanism-head error, we re-evaluated the three validation cohorts with the mechanism-derived rules (i) removed and (ii) fired in the wrong direction—an adversarial worst-case scenario in which every mechanism call was inverted—and recomputed the area under the receiver operating characteristic curve (AUC) and sensitivity (Supplementary Figure S7B).

### Bayesian aggregation and final classification

Rule and language-model strengths were combined under the Tavtigian Bayesian framework (Tavtigian et al., 2018; Pejaver et al., 2022). Each rule strength *s* was mapped to a likelihood ratio *λ* using an exponential table parameterized by a single very-strong odds *O_PV*:

λsupporting=OPV18,λmoderate=OPV14,λstrong=OPV12,λvery−strong=OPV.

For pathogenic-direction rules, the likelihood ratio is greater than 1; for benign-direction rules, its reciprocal is used. Given a prior probability of pathogenicity *π* = 0.1, the posterior *p* satisfies $\log\left(p\;/\;\left(1-p\right)\right)=\log\left(\pi \;/\;\left(1-\pi \right)\right)+\Sigma ᵢ\;\log\left(\lambda ᵢ\right)$ . The posterior was mapped to the five ACMG/AMP classes using published thresholds (Richards et al., 2015; Pejaver et al., 2022). This additive combination of log-likelihood ratios adopts the conditional-independence assumption of the published ACMG/AMP Bayesian point system (Tavtigian et al., 2018; Pejaver et al., 2022), the framework used across ClinGen Variant Curation Expert Panel implementations. The evidence types entering the aggregation draw on largely distinct data modalities—population-frequency (BA1/BS1), functional (PS3/BS3), and case-level (PS4)—and the combined strength of purely computational criteria is capped under the ACMG/AMP specifications (Richards et al., 2015; Biesecker et al., 2018), which bounds the influence of any residual correlation among the predictor-based rules. A side branch in the pipeline bypassed the language-model layer when the rule-engine posterior was already ≥ 0.99 or ≤ 0.10, conserving language-model calls for unambiguous variants.

### Decision-curve analysis

For each tool, we computed net benefit (Vickers and Elkin, 2006) across decision thresholds *t* in the range [0.01, 0.50]. Non-probability predictor scores were first calibrated to a probability scale via five-fold cross-validated isotonic regression (Niculescu-Mizil and Caruana, 2005) on the held-out cohort. All tools were evaluated on the 829-variant common-coverage cohort to ensure a matched comparison.

### Mechanism-aware paired comparison

A paired set of 1,930 variants of uncertain significance was drawn from the three validation cohorts (343 from the post-2024 ClinVar cohort, 62 from the *OTOF* cohort, and 1,525 from the DVD cohort). Each variant was evaluated twice under identical five-source retrieval: once with mechanism-aware prompting and once with the generic-prompt control. For the strength-as-classifier analysis, evidence strengths were mapped to numeric scores, and a per-variant pathogenic-direction score was computed as *PS3 + PS4 − BS3*. Areas under the receiver-operating curve and their differences between the two conditions were estimated using a 1,000-resample paired bootstrap.

### Statistical analysis

AUCs were reported with bootstrap 95% confidence intervals from 1,000 stratified resamples (DiCiccio and Efron, 1996). Proportions were reported with Wilson 95% confidence intervals (Wilson, 1927). Calibration of the OtoVCE posterior probability was summarized using the Brier score (Brier, 1950) and the expected calibration error. Three-class agreement between OtoVCE and the ClinGen Hearing Loss VCEP expert curation was quantified using Cohen’s κ (Cohen, 1960; Landis and Koch, 1977), reported with a bootstrap 95% confidence interval (10,000 resamples). All random operations used a fixed seed.

### Computational cost

In a sample of 40 uncertain variants routed to the language-model evidence layer, we recorded per-variant wall-clock time separately for literature retrieval and the language-model call, together with DeepSeek-V3 token usage, and computed API costs at the DeepSeek list price.

### ClinVar external audit

For each of the nine high-consensus disagreement candidates, we queried NCBI E-utilities and retrieved the current ClinVar aggregate classification, review status, and number of supporting submissions.

## Results

### Framework design

**FIGURE 2:**
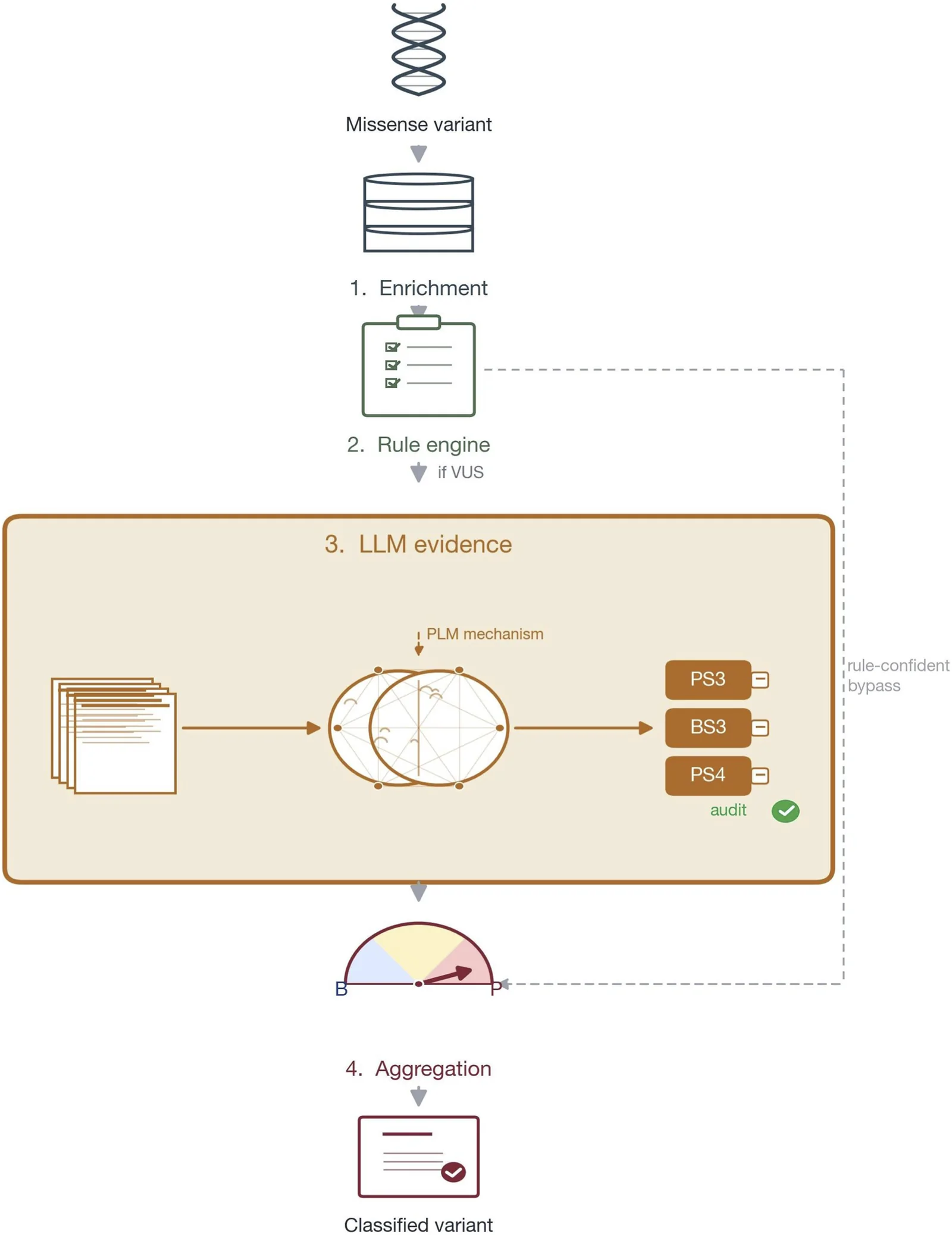
Architecture of the OtoVCE framework. Four-stage pipeline for classification of a hearing-loss missense variant: multi-source enrichment, the ClinGen Hearing Loss VCEP rule engine, a mechanism-aware language-model evidence layer (which retrieves the literature from five sources and prompts a language model under the Brnich functional-evidence rubric with the predicted disease mechanism as context), and a Bayesian aggregation step producing the five ACMG/AMP classes. Variants classified with high confidence by the rule engine bypass the language-model evidence layer.

OtoVCE realizes this design as a four-stage framework (Figure 2), in which the language model acts as a structured evidence extractor within a calibrated ACMG/AMP rule engine, rather than as an end-to-end classifier. Rule-encodable evidence—six *in silico* missense predictors, gnomAD allele frequencies, UniProt domain annotations, and a hearing-loss-specific multi-task protein language model with a pathogenicity head and a mechanism head—is first aggregated by the ClinGen Hearing Loss VCEP rule set (Oza et al., 2018) into a preliminary ACMG/AMP class (Tavtigian et al., 2018). Variants that the rule engine leaves uncertain are passed to a mechanism-aware literature evidence layer. This layer retrieves the literature from five sources, prompts the language model (DeepSeek-AI, 2024) under the Brnich rubric (Brnich et al., 2020) with the predicted disease mechanism as context, and returns Brnich-graded PS3, BS3, and PS4 strength assignments with PubMed-verified citations. Rule and language-model strengths are then combined into a posterior probability mapped to the five ACMG/AMP classes (Pejaver et al., 2022).

Variants for which the rule engine has produced a confident classification bypass the language-model layer, restricting its invocation to variants that remain uncertain.

### Sensitivity at low false-positive rates

**FIGURE 3:**
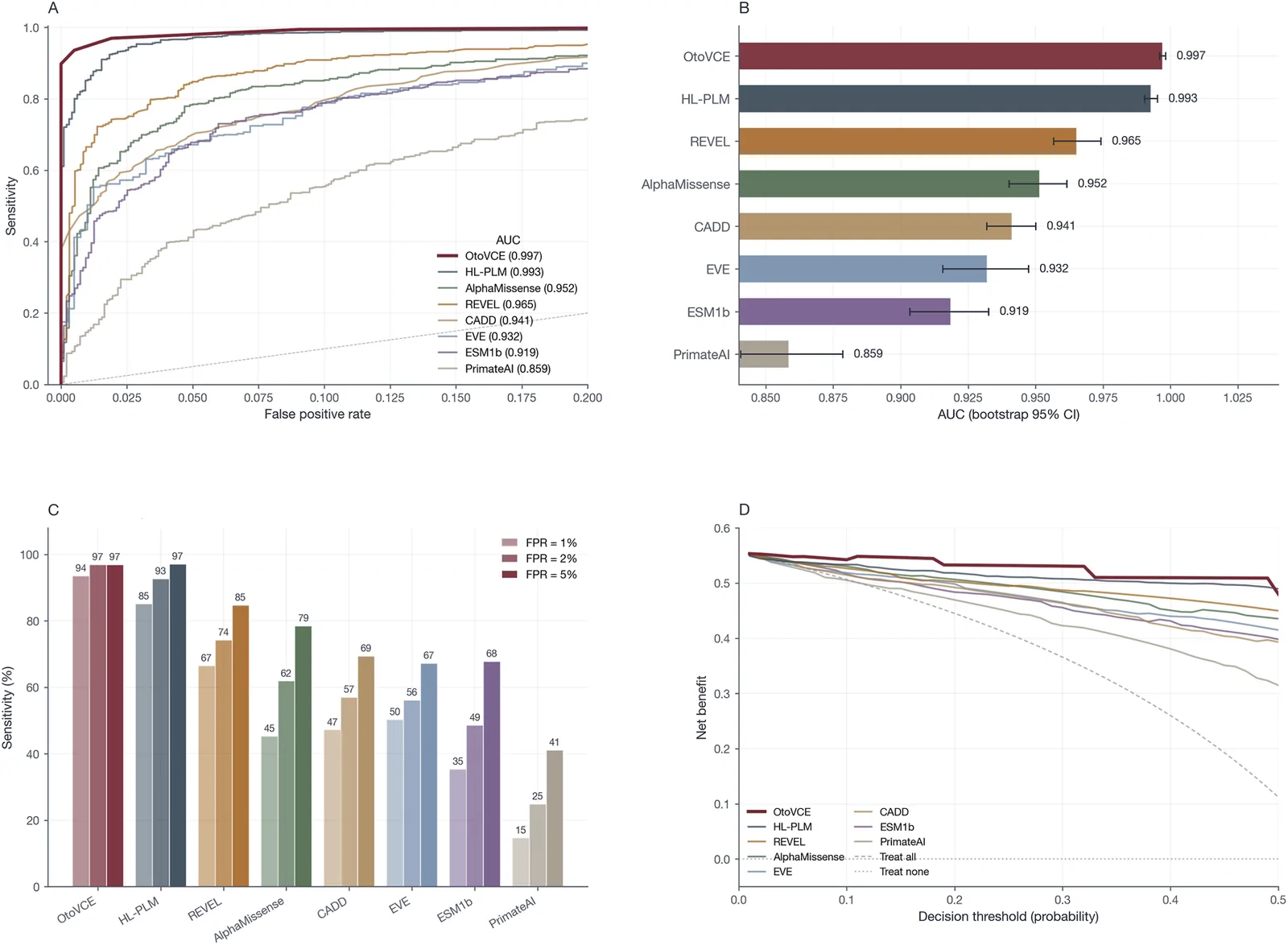
Classification performance on the post-2024 ClinVar cohort (N = 1,885). **(A)** Receiver-operating characteristic curves for OtoVCE and seven baseline tools, zoomed to false-positive rates of 0.20 or below. **(B)** Area under the receiver-operating curve with bootstrap 95% confidence intervals (1,000 stratified resamples). **(C)** Sensitivity at false-positive rates of 1%, 2%, and 5%. **(D)** Decision-curve analysis on the 829-variant common-coverage subset.

The first of the four cohorts—the held-out post-2024 ClinVar cohort—provides the cleanest test of classification performance under temporal hold-out and is reported first. At a 1% false-positive rate in this cohort (N = 1,885 held-out variants), OtoVCE recovered 93.6% of pathogenic and likely-pathogenic variants, compared with 45.4% for AlphaMissense (Cheng et al., 2023) and 66.5% for the strongest individual predictor, REVEL (Ioannidis et al., 2016) (Figure 3C; Table 1).

OtoVCE achieved an AUC of 0.997, and its 95% confidence interval (DiCiccio and Efron, 1996) did not overlap that of the next-best baseline at 0.993 (Figures 3A,B). Restricting the comparison to the 829 variants scored using every tool preserved this margin. Decision-curve analysis (Vickers and Elkin, 2006) (Figure 3D) extended this finding to clinical-utility space: OtoVCE was the only tool among eight to remain above the treat-all reference at every decision threshold tested.

### Generalization under the distribution shift

**FIGURE 4:**
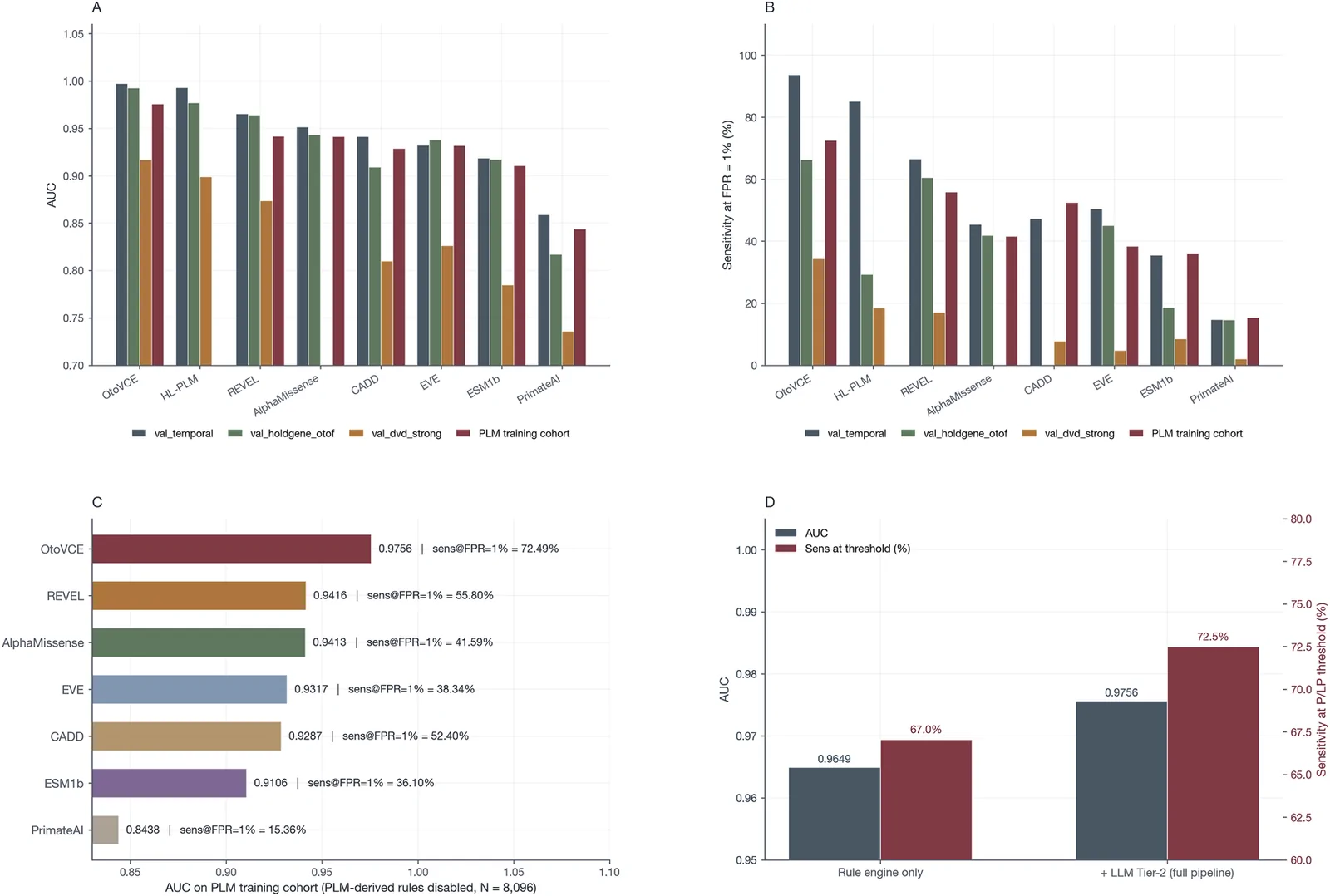
Generalization across cohorts and memorization defense. **(A)** Area under the receiver-operating curve for OtoVCE and seven baselines on the four validation cohorts. **(B)** Sensitivity at a 1% false-positive rate on the same cohorts. **(C)** Per-tool AUC and sensitivity at a 1% false-positive rate for OtoVCE and six individual predictors on the protein-language-model training cohort, evaluated with the two protein-language-model-derived ACMG rules disabled. **(D)** Stage-by-stage contribution of the rule engine and the language-model evidence layer on the same cohort.

The AUC of 0.997 reported above establishes classification performance under temporal hold-out, but performance on any single cohort may still reflect overfitting to its curation style. To probe generalization, the framework was re-evaluated under three additional sources of distribution shift (Figures 4A,B; Table 1).

We withheld the entire *OTOF* gene from protein language model training and evaluated on every ClinVar variant in *OTOF*. The framework’s lead was preserved at an AUC of 0.993. On the Deafness Variation Database (Azaiez et al., 2018), absolute performance declined uniformly across all tools; however, the framework retained its lead at an AUC of 0.917. Finally, we re-evaluated the framework on the protein-language-model training cohort, with the two ACMG rules drawing signals from that model disabled; any residual advantage must therefore originate from the rule engine or the literature-evidence layer. Under this configuration, the framework achieved an AUC of 0.976 and a sensitivity of 72.5% at the 1% false-positive rate, exceeding every individual predictor (Figure 4C; Table 1). Decomposing the pipeline (Figure 4D), the rule engine alone reached an AUC of 0.965; adding the literature-evidence layer increased this to 0.976 and increased sensitivity by 5.5 percentage points.

### The mechanism cue drives the evidence-layer increase

The pipeline decomposition above attributes a 5.5-percentage-point sensitivity increase to the literature-evidence layer. To isolate the contribution of the mechanism cue, we conducted a paired comparison on 1,930 uncertain variants drawn from the three validation cohorts, in which retrieval, model, and rubric were held constant and only the mechanism cue was toggled on or off (Figure 1). Relative to the generic prompt, the model cited 7.1-fold more PubMed identifiers per variant (Figure 1A) and extracted 11.6-fold more quantitative evidence (Figure 1B). At moderate-or-stronger strengths, mechanism-aware prompting yielded 2.1-fold more PS3 assignments, 8.8-fold more PS4 assignments, and each of the cohort’s seven BS3 ≥ moderate assignments (Figure 1C).

We evaluated whether these output-volume differences reflected genuine accuracy or verbosity alone by treating the model’s evidence strengths as a stand-alone classifier of pathogenicity. Under mechanism-aware prompting, the strength score reached an AUC of 0.715; under the generic prompt, AUC decreased to 0.537 (Figure 1D; paired-bootstrap ΔAUC = 0.18, 95% CI = 0.16–0.20). Each of the 3,185 PubMed identifiers cited by the model across the three validation cohorts resolved to an indexed PubMed record (Supplementary Figure S1B).

The mechanism head reached 87.6% top-1 accuracy (95% CI = 81.8%–92.7%; N = 137 wet-laboratory-labeled variants) against curated functional mechanisms, increasing to 97.8% (95% CI = 94.9%–100%) at the loss-of-function-type-versus-dominant-negative granularity consumed by the phenotype-mechanism rule; the residual confusions fell within the loss-of-function super-class and left unchanged the inheritance signal on which the rule engine acts (Supplementary Figure S7A). The final classification was moreover robust to the mechanism-head error: under an adversarial configuration in which every mechanism call was flipped to the wrong direction, AUC decreased by at most 0.030 units across the three cohorts (post-2024 ClinVar 0.997 → 0.991; *OTOF* 0.993 → 0.992; DVD 0.917 → 0.887; Supplementary Figure S7B) because the mechanism head enters aggregation only as supporting-to-moderate-strength evidence within the seventeen-rule Bayesian framework.

### Clinical actionability and expert concordance

**FIGURE 5:**
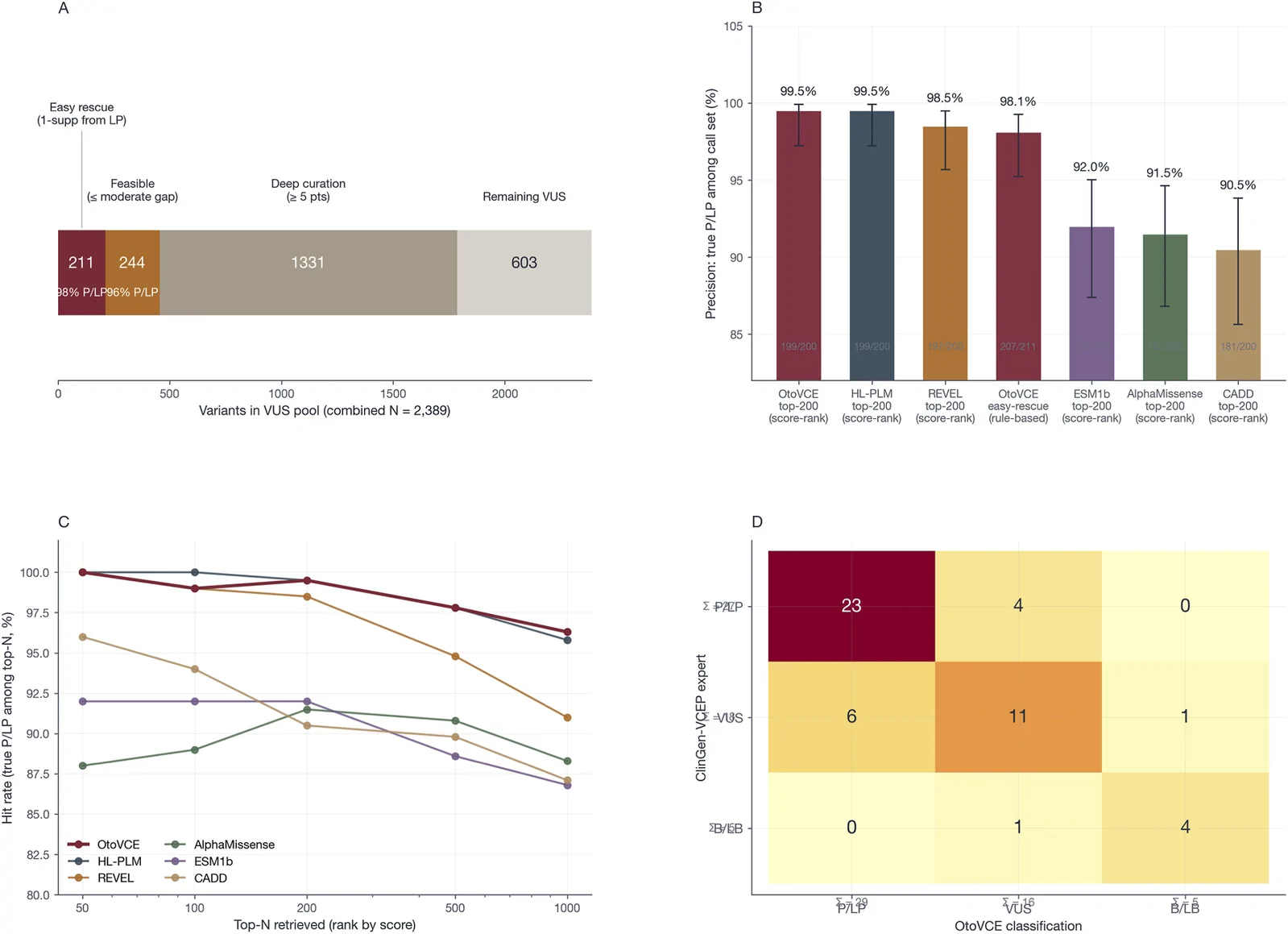
Clinical workflow integration and concordance with expert curation. **(A)** Triage of the 2,389 variants of uncertain significance pooled from the three validation cohorts into four tiers (*easy rescue*, *feasible*, *deep curation*, and *remaining VUS*). **(B)** Precision of the easy-rescue tier compared with the top-200 score-ranked picks of leading individual predictors on the same pool, with Wilson 95% confidence intervals. **(C)** Top-N hit-rate for each tool across retrieval depths 50–1,000. **(D)** Three-class concordance with ClinGen Hearing Loss VCEP curation on the 50 expert-curated variants in our cohorts; Cohen’s κ = 0.57 (95% bootstrap CI = 0.34–0.77).

Across the three validation cohorts, the rule engine returned 2,389 variants as uncertain. The framework stratified this pool by distance to the likely-pathogenic threshold (Figure 5A). Of the 2,389 variants, 211 fell one piece of supporting evidence short of the likely-pathogenic threshold—the *easy-rescue* tier—and 207 of these 211 variants (98.1%; Figure 5B) were genuinely pathogenic or likely pathogenic in the cohort labels. Across retrieval depths of 50–1,000 on the same uncertain-variant pool, the framework’s hit rate tracked the strongest individual predictor and exceeded the remaining baselines at every depth (Figure 5C). The marginal cost of the evidence layer is negligible—a mean USD 0.0004 and a 2.4-s language-model call per interpreted variant—and is incurred only for the uncertain minority routed to it (see *Computational cost*).

Fifty variants in our cohorts were curated using the ClinGen Hearing Loss VCEP. The framework agreed with the expert panel at Cohen’s κ = 0.57 (95% bootstrap CI = 0.34–0.77) across the three ACMG tiers; the framework and the panel agreed in direction (pathogenic versus benign) on all 27 variants assigned a definitive class by both, with no benign–pathogenic reversals (Cohen, 1960; Landis and Koch, 1977) (Figure 5D). No variant classified as benign or likely benign by the expert panel was reclassified as pathogenic by the framework.

### Disagreement analysis and failure modes

**FIGURE 6:**
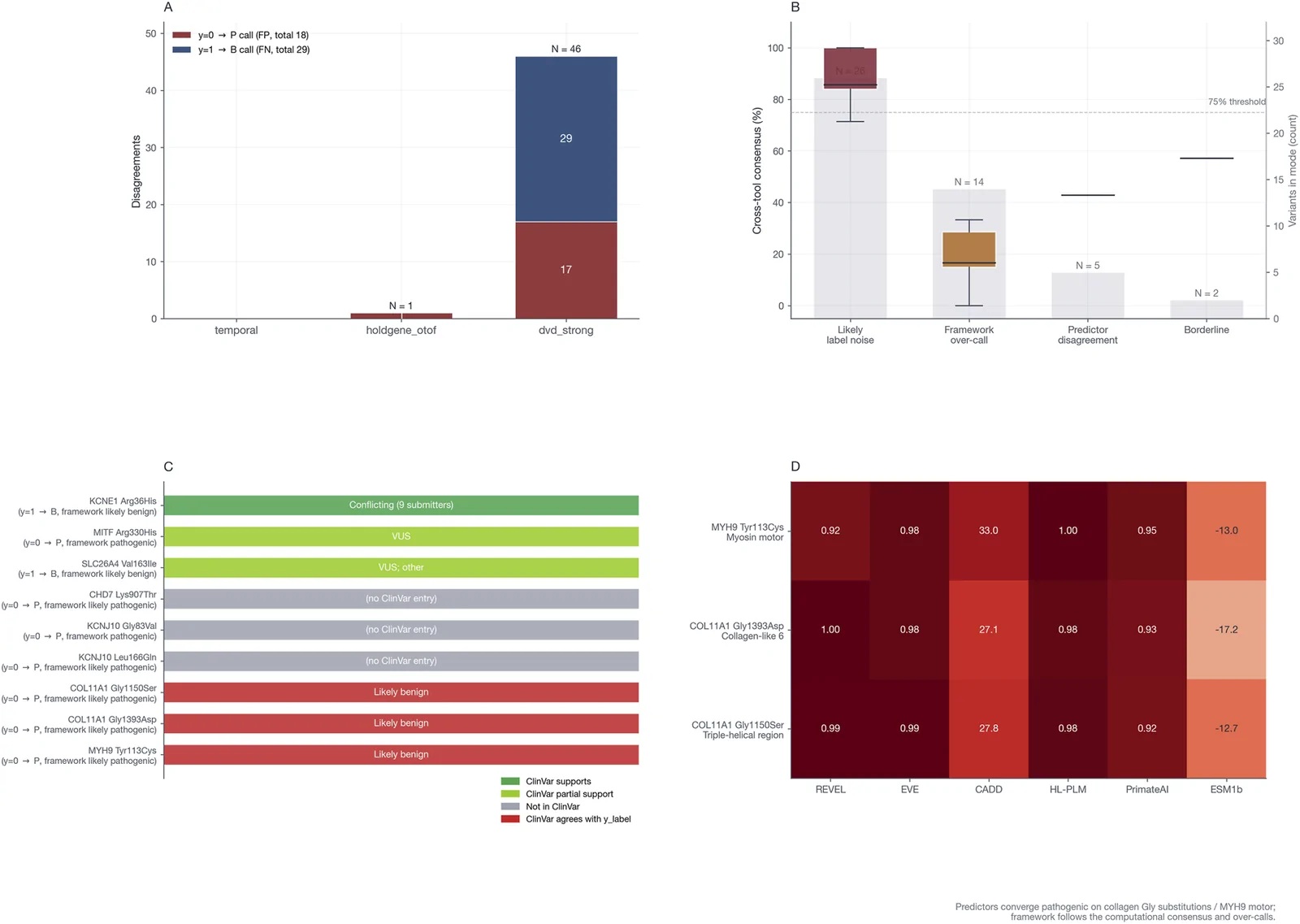
Disagreement analysis identifies label-noise candidates and a structural over-call failure mode. **(A)** Forty-seven variants for which OtoVCE’s final class differed from the cohort label, broken down by cohort and direction of disagreement. **(B)** Failure-mode taxonomy with box-and-whisker plots of cross-tool consensus within each mode. **(C)** ClinVar external audit of the nine variants reaching 100% cross-tool consensus with at least five predictors scored. **(D)** Predictor profiles of the three ClinVar-contradicted variants.

Across the 4,925 variants in the three validation cohorts, the framework’s final class differed from the cohort label on 47 variants. Forty-six of these disagreements were on the Deafness Variation Database cohort (Figure 6A). Twenty-six of the 47 reached cross-tool consensus of at least 75% with the framework (Figure 6B), a pattern consistent with label noise; fourteen showed the opposite pattern (predictor agreement ≤33%) and were attributed to framework overcalls. The remaining seven variants split between predictor disagreement (N = 5) and borderline assignments at the rule-engine threshold (N = 2).

External adjudication on the nine high-consensus candidates against current ClinVar status (Figure 6C) corroborated three (*KCNE1* p. Arg36His, *MITF* p. Arg330His, and *SLC26A4* p. Val163Ile), contradicted three (two *COL11A1* Gly → X substitutions and a *MYH9* p.Tyr113Cys—the framework’s documented overcalls), and left three without a ClinVar record. The three contradictory variants share an interpretable structural pattern (Figure 6D): every available *in silico* predictor reaches or approaches its pathogenic ceiling for collagen Gly → Asp/Gly → Ser substitutions and for the *MYH9* myosin-motor-domain Tyr → Cys substitutions.

## Discussion

The present study positions a large language model as a structured evidence extractor within an otherwise conventional ACMG/AMP rule engine. This role is distinct from the more common use of foundation models as end-to-end classifiers in clinical and biomedical settings (Singhal et al., 2023). Three aspects of our results merit further discussion: the nature of the literature-evidence layer’s contribution, the basis for the effect of mechanism-aware prompting, and the implications of the framework’s failure modes for sequence-based predictors as a class.

The numerical gain attributable to the language-model evidence layer is modest: 0.011 AUC units and 5.5 percentage points of sensitivity at the framework’s posterior threshold. This magnitude is comparable to the gap between any two leading missense predictors on the ClinGen calibration scale (Pejaver et al., 2022) and would not, in isolation, justify a new framework. We interpret the contribution as structural rather than statistical. A continuous-score predictor leaves the categorical decision to a downstream curator (Ioannidis et al., 2016; Cheng et al., 2023). A rule-based ACMG/AMP framework returns a class but operates only on evidence that can be programmatically encoded (Li and Wang, 2017; Kopanos et al., 2019). A single-shot language-model classifier may have accessed the relevant literature but produces unstructured output with citations that are difficult to verify. The literature-evidence layer occupies the intersection between these approaches: variants reclassified through this layer acquire Brnich-graded strength assignments supported by audited PubMed citations (Supplementary Figure S1B), which are the form of input the ACMG/AMP framework was designed to integrate.

A central finding is that a single prompt cue—the predicted disease mechanism—shifted the language model from a regime no better than chance (AUC = 0.537) to a discriminative classifier (AUC = 0.715) on identical retrieved literature (Figure 1). The present experiment does not resolve why the cue has this effect. Three non-mutually exclusive explanations are plausible: the cue may function as an attention anchor (Vaswani et al., 2017) that directs the model toward biophysically relevant passages; it may prime the model’s vocabulary toward mechanism-specific terms that subsequently surface relevant articles; or it may interact with the Brnich rubric itself, refining the mapping between the literature and strength levels. Distinguishing between these explanations is an open question for future work. Our finding is consistent with prior work on retrieval-augmented language model applications in scientific text processing (Lewis et al., 2020; Singhal et al., 2023; Lala et al., 2023), which shows that a small domain-specific context cue can produce measurable and rubric-calibrated changes in model output. Our setting differs in that the cue is generated upstream by a separate model (Brandes et al., 2023) rather than supplied by a human annotator.

The framework’s most informative failure mode is the over-call on the three variants contradicted by ClinVar (Figure 6D). All three variants reach the pathogenic ceiling of every available *in silico* predictor (Frazer et al., 2021; Cheng et al., 2023; Brandes et al., 2023), yet ClinVar concurs with the original benign assignment. We interpret this as a structural property of sequence-based predictors as a class: their clinical benignity depends on information inaccessible to any sequence-only model, including penetrance in the carrying family, location in a buffered structural region, or compensatory expression from alternative isoforms. Any framework aggregating such predictors will inherit the same ceiling. The ACMG/AMP specifications already anticipate this limitation by capping the combined strength of purely computational evidence (Richards et al., 2015; Biesecker et al., 2018).

Beyond the diagnostic-accuracy and failure-mode findings above, the framework’s contribution to the clinical-laboratory workflow is modest but specific (Figure 5). The *easy-rescue* tier reaches 98.1% precision on 211 variants, comparable to the top-200 score-ranked picks of any leading individual predictor. The distinction lies in the form of the output: OtoVCE returns a categorical recommendation that maps directly onto a curator’s decision to pursue functional or segregation follow-up, whereas leading predictors return continuous scores for which the cut-off must be independently determined by the laboratory.

Circularity is an intrinsic consideration for any framework that aggregates multiple pre-classified evidence sources: because the reference labels, the protein-language-model training targets, the ClinVar table underpinning PS1 and PM5, and a portion of the extracted PS3/BS3/PS4 literature can trace to overlapping curatorial sources, such a model could, in principle, appear accurate by re-reading its own labels rather than by weighing evidence. We anticipated this failure mode and controlled for it by construction: each of the four cohorts defined in the *Materials and Methods* was assembled to sever one specific link in the label-to-evidence chain. The post-2024 ClinVar cohort enforces a temporal break—the protein language model was trained only on labels curated before 1 January 2024, and the cohort was held out for the entire development process. The *OTOF* cohort enforces a gene-level break, with the entire gene withheld from protein language model training (AUC = 0.993). The Deafness Variation Database cohort provides a provenance break, drawing on curatorial sources distinct from those used in the ClinVar-derived cohorts (AUC = 0.917). Most directly, re-evaluating the framework on the protein-language-model training cohort with the two protein-language-model-derived ACMG rules disabled removes the one path by which memorized training labels could inflate performance; the framework nonetheless reached an AUC of 0.976, so any residual advantage must originate from the rule engine or the literature-evidence layer rather than from label recall. Two further design choices bound the concern by construction: PS1 and PM5 use ClinVar solely as a transparent, deterministic reference table of already-classified variants, and each of the 3,185 PubMed identifiers cited by the evidence layer resolved to an indexed PubMed record (Supplementary Figure S1B), confirming that the extracted evidence is the auditable primary literature rather than a re-encoding of the label.

Several practical limitations bound these conclusions. First, label noise is persistent in hearing-loss missense benchmarks (Harrison et al., 2017); our reported metrics should, therefore, be interpreted as a lower bound on agreement with future-revised labels. Second, the evidence layer is dependent on the literature surfaced by its retrieval sources (Allot et al., 2023); variants in genes with the sparse functional literature remain uncertain after the literature-evidence step. Third, domain-specificity is, by design, confined to three well-defined, swappable components rather than distributed across the pipeline: the ClinGen Hearing Loss VCEP rule set (Oza et al., 2018), the hearing-loss-specific protein language model and its mechanism head, and the curated hearing-loss functional-assay whitelist. The domain-agnostic machinery—five-source literature retrieval, the Brnich functional-evidence rubric (Brnich et al., 2020), Tavtigian Bayesian aggregation (Tavtigian et al., 2018), and the PubMed-verified citation audit—carries over unchanged to any Mendelian disease. Porting OtoVCE to a new domain, therefore, reduces to substituting these three elements: the target disease’s VCEP specification for the hearing loss VCEP rule set; a domain-specific protein language model or mechanism ontology for the hearing-loss model and its mechanism head; and the target domain’s assay list for the hearing-loss functional-assay whitelist. The one domain-specific input is a mechanism vocabulary and a labeled mechanism set with which to train and validate the mechanism head, analogous to the labeled hearing-loss mechanism set used here. Hereditary cardiomyopathy is a well-posed first target for exactly this reason: an established ClinGen Cardiomyopathy VCEP specification is already published, and its sarcomeric mechanisms—for instance, gain-of-function versus loss-of-function modes—are sufficiently well characterized to supply the mechanism vocabulary the mechanism head requires. Two concrete extensions follow: a segregation-evidence extractor for the PP1 rule, analogous to the present PS3/BS3/PS4 extraction; and a gating rule requiring at least one piece of non-computational evidence on structural regions where predictors converge, which would mitigate the over-call mode.

The framework is architected so that the base language model’s pre-training corpus cannot constitute a leakage pathway. The model operates as a structured evidence extractor, not a classifier: it never emits an ACMG/AMP class but returns Brnich-graded PS3, BS3, and PS4 strengths, each anchored to a specific PubMed identifier that is verified against NCBI E-utilities and removed if it does not resolve (all 3,185 cited identifiers resolved to indexed records; Supplementary Figure S1B). A classification recalled from pre-training therefore cannot enter the pipeline as a class; only a resolvable citation supporting an extractable functional result contributes, and the rule engine assigns the final class. Consistent with this design, the framework’s lead is retained not only under the strictly post-2024 temporal hold-out but under gene-level leave-out (*OTOF* cohort, AUC = 0.993) and external-provenance shift (DVD cohort, AUC = 0.917), where memorization of the training distribution cannot account for performance.

The present study establishes, in the hearing-loss setting, that a language model used as a structured evidence extractor rather than as a classifier can serve as a complementary component within a calibrated ACMG/AMP workflow.

## Data Availability

The four validation cohorts used in this study are derived from publicly available sources. The post-2024 temporal ClinVar cohort (N = 1,885) and the *OTOF*-gene held-out cohort (N = 205) are derived from ClinVar releases (https://ftp.ncbi.nlm.nih.gov/pub/clinvar/). The Deafness Variation Database strong-evidence cohort (N = 2,835) is derived from the Deafness Variation Database v9 (https://deafnessvariationdatabase.org). The processed CSVs for all four cohorts, the per-variant predictions (including the hearing-loss-specific protein language model’s pathogenicity-head and mechanism-head outputs), the ACMG/AMP rule firings, and the LLM-extracted evidence, together with the 3,185 cited PubMed identifiers and the paired-A/B prompt outputs, are deposited in Zenodo (DOI: 10.5281/zenodo.21245197; archived via the GitHub–Zenodo integration of https://github.com/pipi0616/OtoVCE) under a CC BY 4.0 license.

## Supplementary material

SUPPLEMENTARY FIGURE S7 Mechanism-head validation and robustness of the final classification to mechanism error. **(A)** Confusion matrix of the protein-language-model mechanism head against 137 wet-lab-curated mechanisms, with 4-class and loss-type-vs-dominant-negative axis accuracies. **(B)** Final-classification AUC on the three validation cohorts under the mechanism rule full, removed, and adversarially flipped.
