# Supplementary material for: OtoVCE: a mechanism-aware language-model evidence layer for hereditary hearing-loss variant interpretation
Source: Front Genet. 2026 Aug 12;17:1880490. doi: 10.3389/fgene.2026.1880490 (PMC13506142; doi:10.3389/fgene.2026.1880490)

Supplementary Figure S2. Inheritance subgroup and per-gene robustness on val_temporal_extended

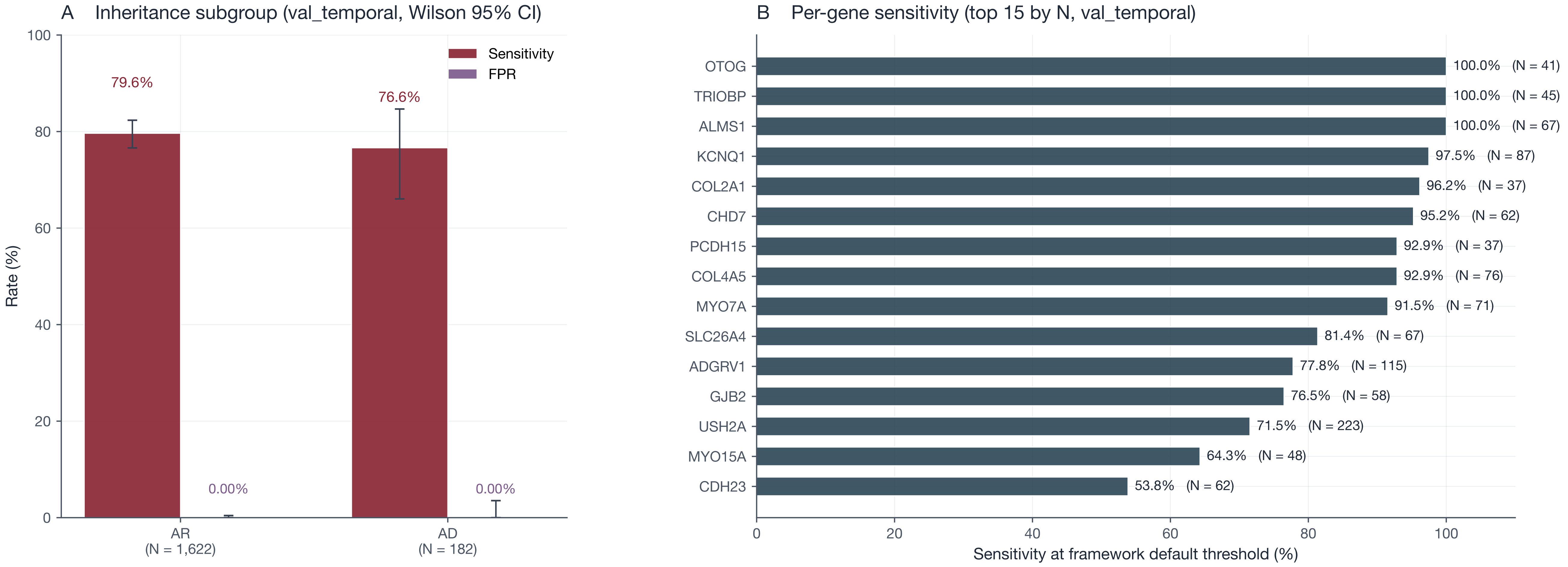

Supplement: Supplementary file 2 [file Image2.pdf]
