# Supplementary material for: OtoVCE: a mechanism-aware language-model evidence layer for hereditary hearing-loss variant interpretation
Source: Front Genet. 2026 Aug 12;17:1880490. doi: 10.3389/fgene.2026.1880490 (PMC13506142; doi:10.3389/fgene.2026.1880490)
Supplement: Supplementary file 4 [file Image4.pdf]

Supplementary Figure S4. Comparison with single-shot LLM baselines and detailed PS4 strength agreement

A OtoVCE vs raw single-shot LLMs across cohorts

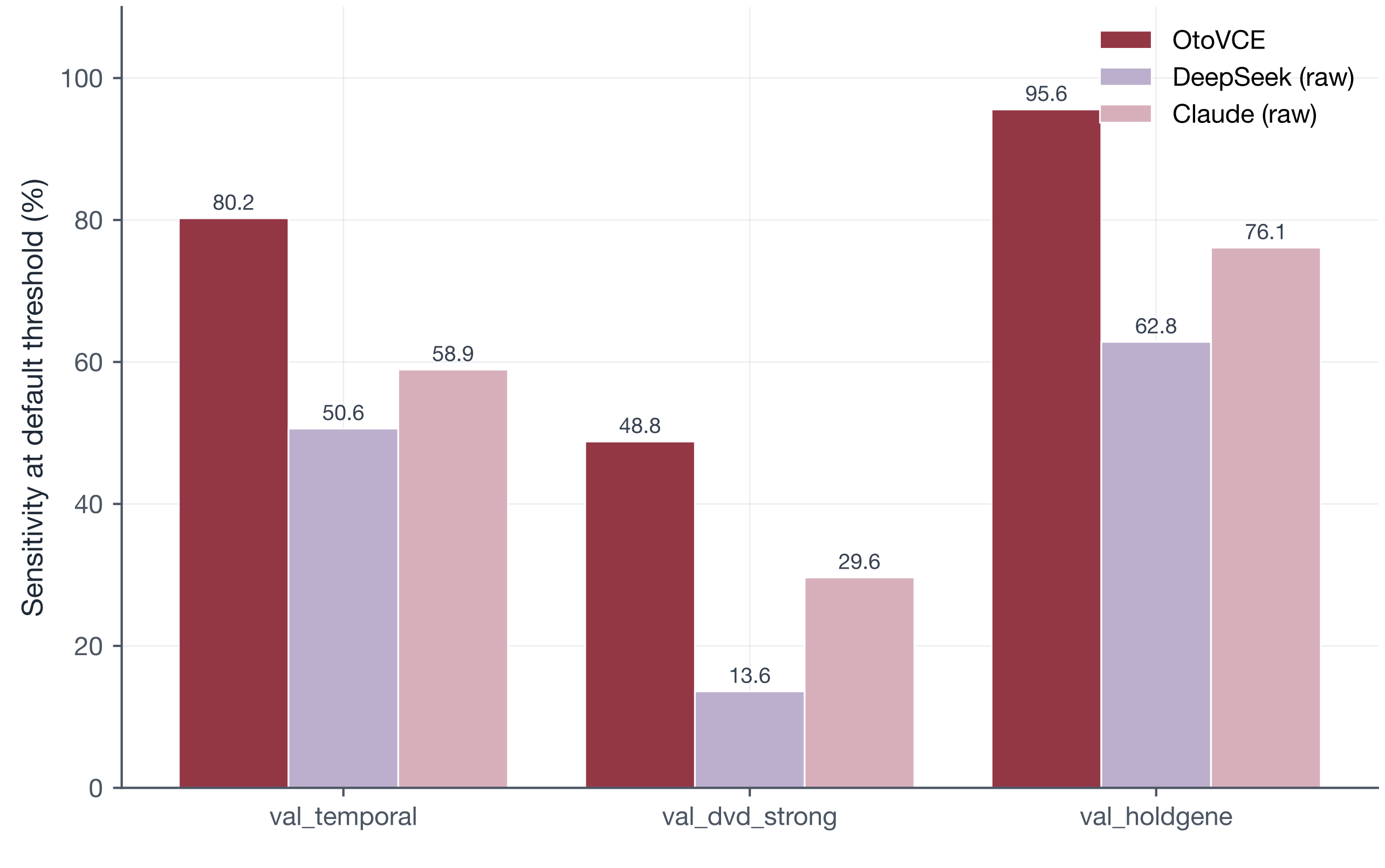

B PS4 strength agreement (paired N = 1,930, 67% diagonal)

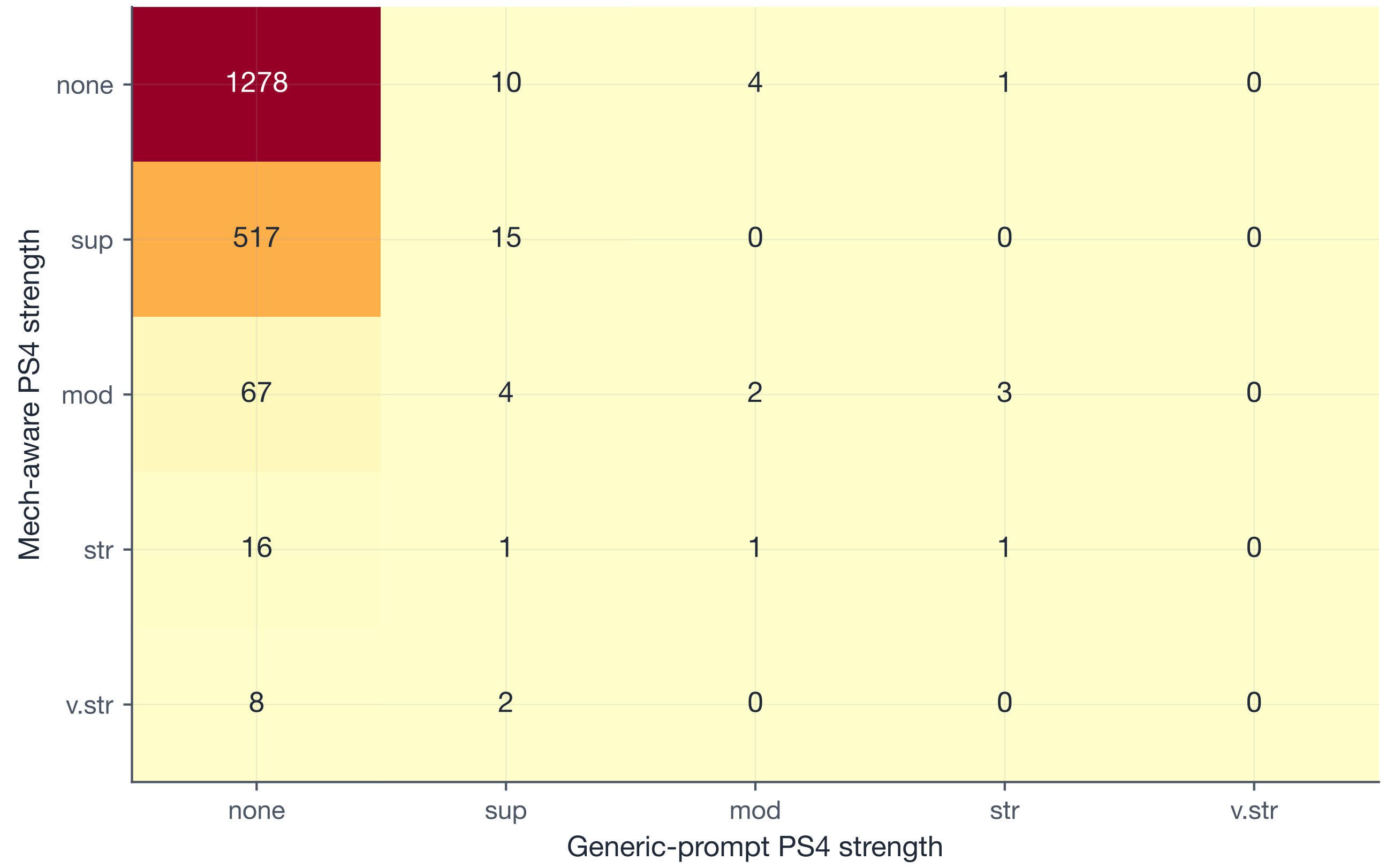

C PMID-per-variant distribution (paired N = 1,930)

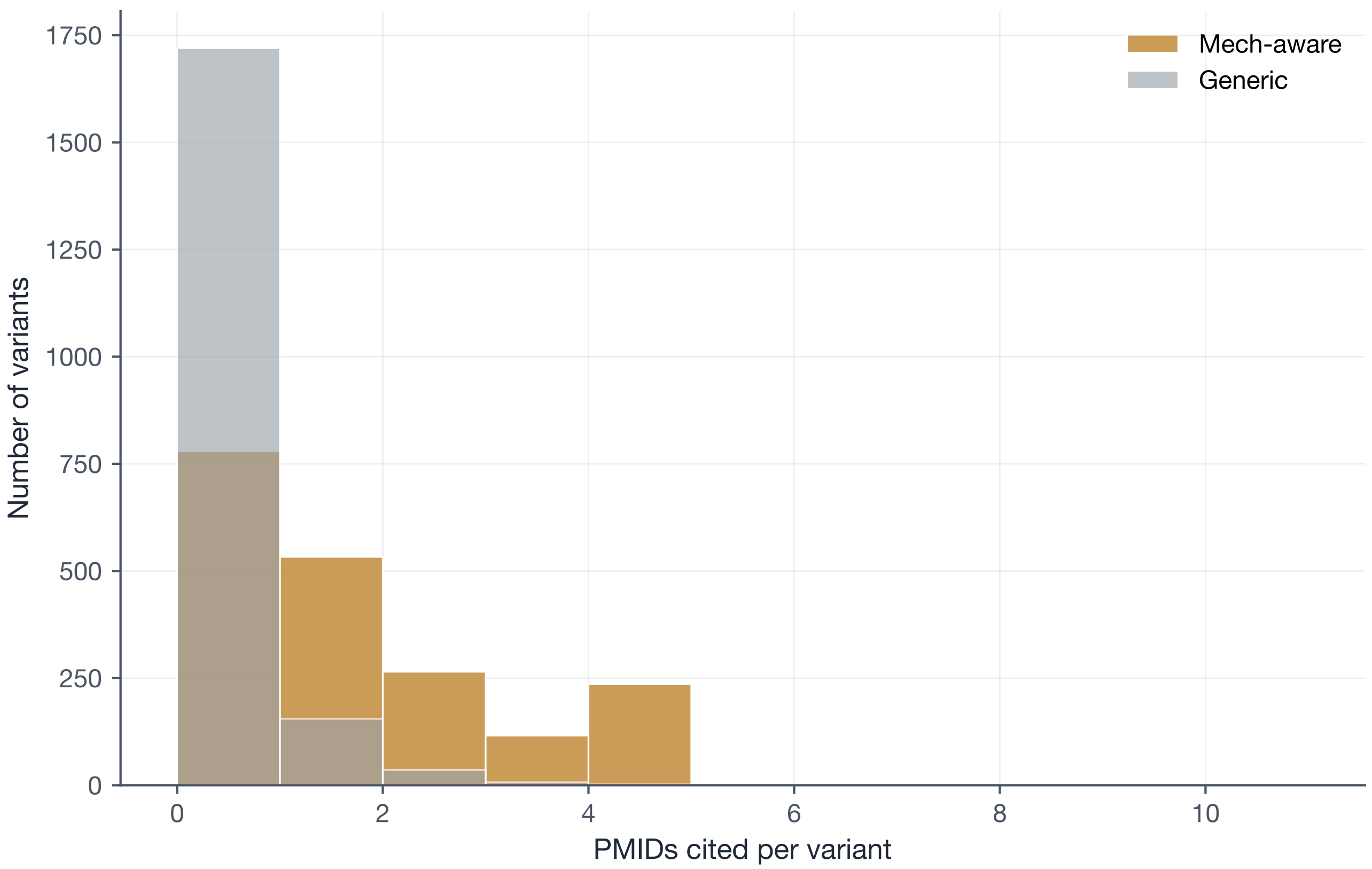

D Worked-example rationale comparison (COL2A1 p.Arg565Cys)

Mech-aware (PS3 = moderate, PS4 = strong; 4 PMIDs):

PMID 38073514 demonstrates that COL2A1 mutants (including p.Arg565Cys) form large aggregates in the ER and elevate ER stress, with wild-type negative control — qualitative loss-of-function in a validated assay (PS3 moderate). For PS4, the variant is reported across 4 PMIDs as a recurrent allele in 11 unrelated Stickler probands.

Generic (PS3 = none, PS4 = none; 0 PMIDs):

No retrieved literature provides functional data or proband counts for the specific variant COL2A1 p.Arg565Cys. The papers focus on Stickler syndrome diagnosis and other COL2A1 mutations.
