# Supplementary material for: OtoVCE: a mechanism-aware language-model evidence layer for hereditary hearing-loss variant interpretation
Source: Front Genet. 2026 Aug 12;17:1880490. doi: 10.3389/fgene.2026.1880490 (PMC13506142; doi:10.3389/fgene.2026.1880490)
Supplement: Supplementary file 5 [file Image5.pdf]

Supplementary Figure S5. AAV-OTOF deployment case study (Chapter D)

A OTOF P/LP variants by AAV-eligibility tier (N = 113)

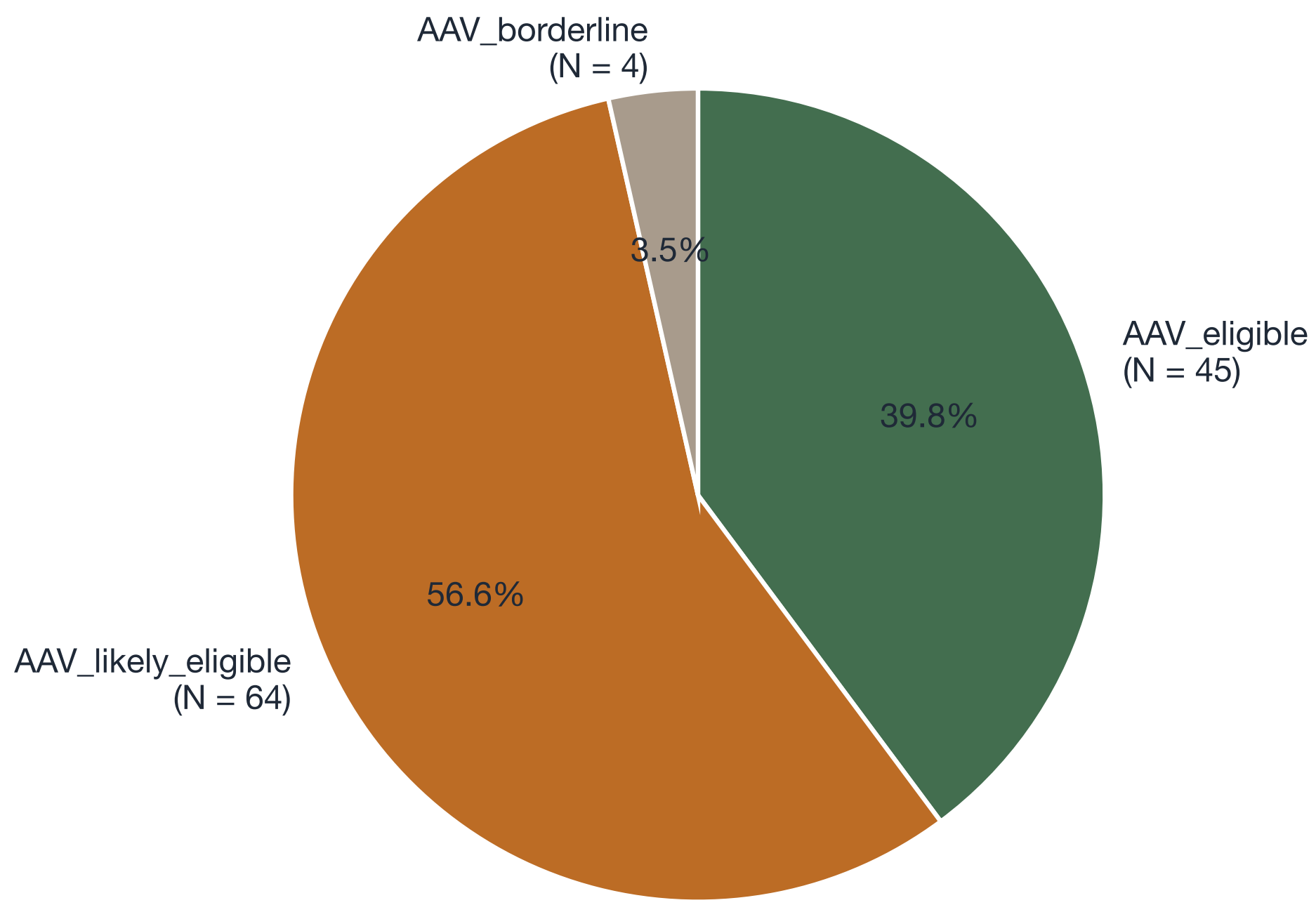

B 10 known DFNB9 trial variants — tool comparison

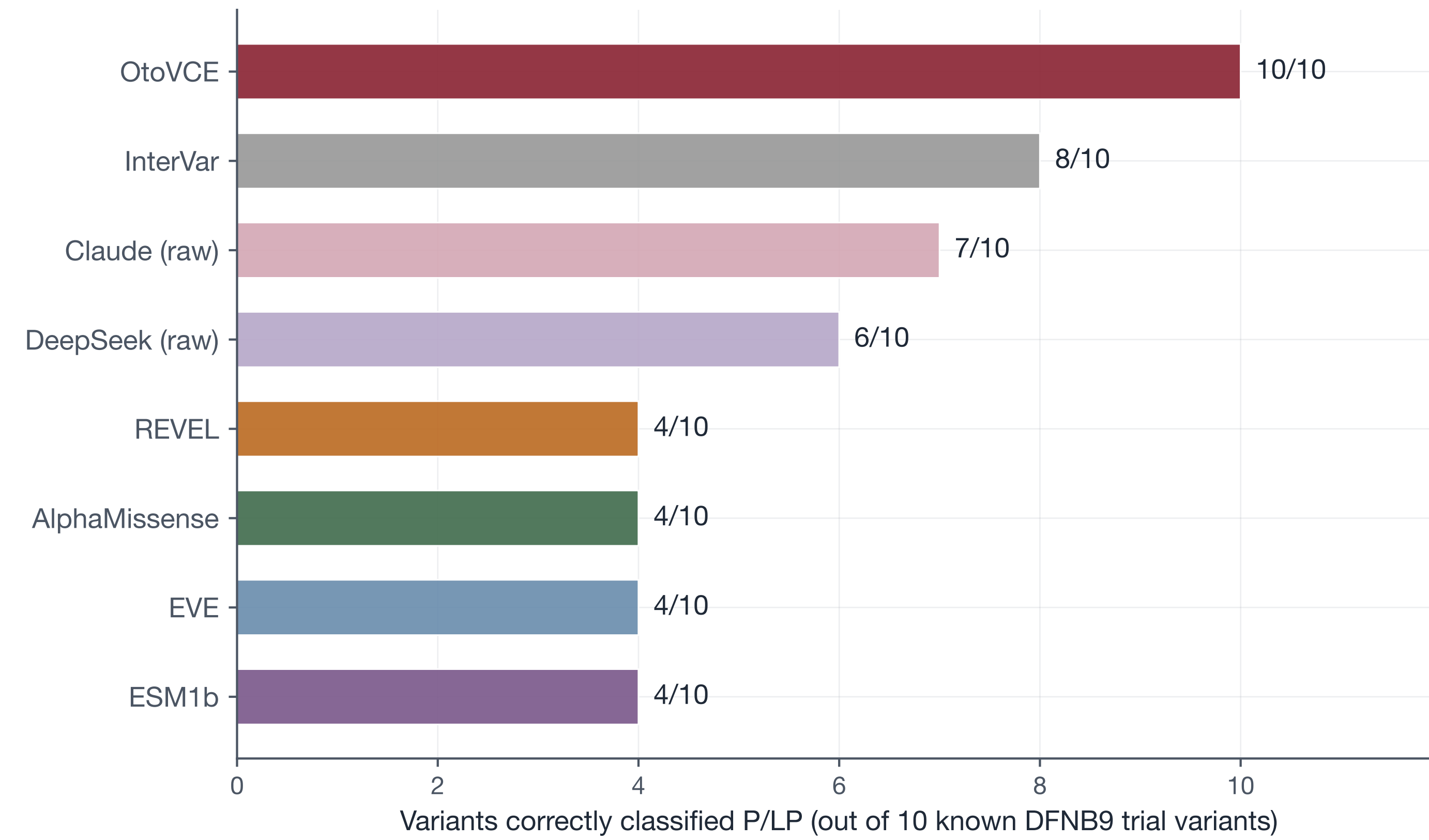

C AAV tier × variant consequence (N = 113 OTOF P/LP)

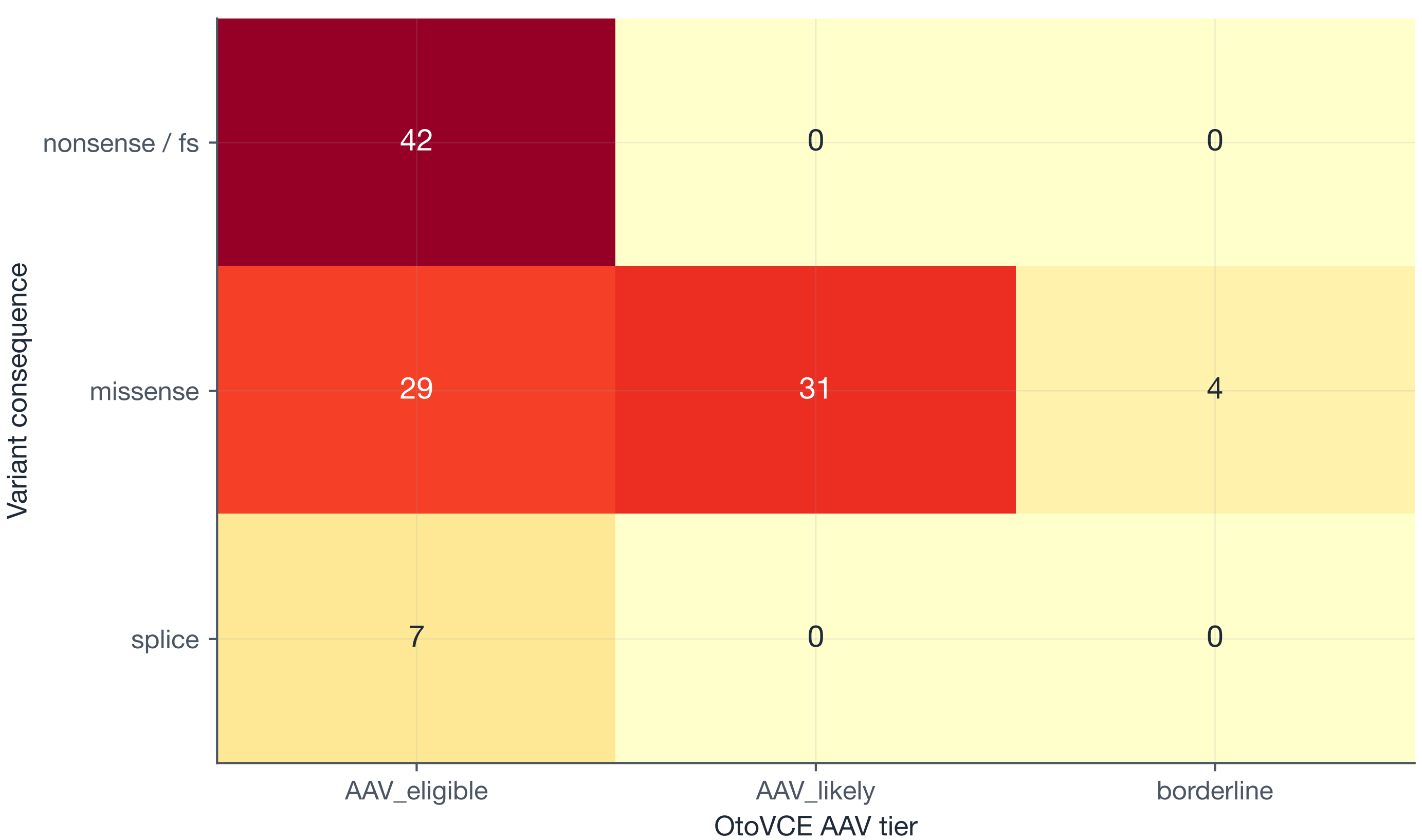

D Illustrative AAV-OTOF case (OTOF p.Pro490Arg, recurrent in clinical trials)

Step 2 — rules engine: VUS (net 3 pts)

Step 3 — multi-agent debate: Likely pathogenic (moderate confidence)

Rationale (excerpt):
C2-3 calcium-binding fold; Pro-to-Arg substitution introduces charge and disrupts the beta-sandwich, mechanistically aligned with otoferlin loss-of-function. Computationally convergent (AlphaMissense 0.979, EVE 0.842, ESM1b strong, HL-PLM 0.979 with LoF as top mechanism head), AF functionally absent in gnomAD.

AAV-OTOF eligibility:
In trans with a second pathogenic allele, this variant supports trial enrollment as biallelic pathogenic genotype.
