# Supplementary material for: OtoVCE: a mechanism-aware language-model evidence layer for hereditary hearing-loss variant interpretation
Source: Front Genet. 2026 Aug 12;17:1880490. doi: 10.3389/fgene.2026.1880490 (PMC13506142; doi:10.3389/fgene.2026.1880490)
Supplement: Supplementary file 7 [file Image7.pdf]

Supplementary Figure S7. Mechanism-head validation and robustness of the final classification to mechanism error

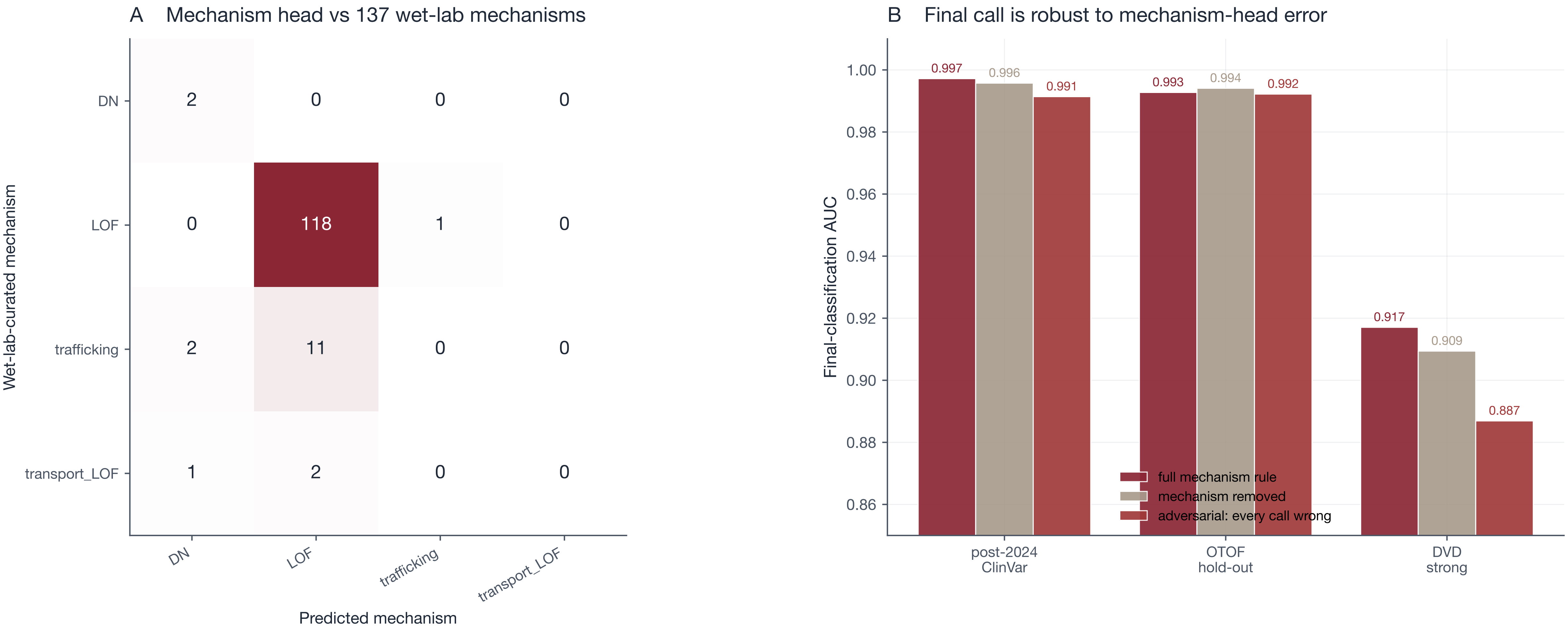

Even under the adversarial worst case (every mechanism call flipped wrong), AUC changes by ≤ 0.030.

4-class accuracy = 87.6% · loss-type vs DN axis (consumed by rule) = 97.8%
